# Evaluation of enamel wear after the use of whitening toothpastes

**DOI:** 10.1007/s00784-026-06855-2

**Published:** 2026-03-31

**Authors:** Antonio José Tôrres Neto, Renata Pedrosa Guimarães, Alexandre Batista Lopes do Nascimento, Hilcia Mezzalira Teixeira

**Affiliations:** 1 Department of Prosthodontics and Oral and Maxillofacial Surgery, Federal University of Pernambuco (UFPE), Recife, PE Brazil; 2 Institute of Science and Technology, São Paulo State University (Unesp), São José dos Campos, Av. Eng. Francisco José Longo, 777 - Jardim São Dimas, São José dos Campos - SP, São Paulo, 12245-000 Brazil

**Keywords:** Dental abrasion, Wear, Whitening, Toothpastes

## Abstract

**Objectives:**

The aim of this study was to evaluate by optical coherence tomography (OCT), the wear of tooth enamel after using different toothpastes for whitening.

**Materials and methods:**

Specimens of bovine teeth, divided into 5 groups (*n* = 10) according to the toothpaste were made: G1 / Oral-B 3D White; G2 / Colgate Total 12 Whitening Gel; G3 / Colgate Luminous White; G4 / White Closeup Now; G5 (control) / distilled water. The OCT images were taken before brushing cycling, with a system operating in the spectral domain, with a center wavelength of 930 nm. The simulated toothbrushing (5000 cycles) was performed with linear movements, under static axial load of 200 g and speed of 4.5 cycles per second, understanding how to cycle the full movement back-and-forth of the toothbrush. New OCT analysis was then performed, and the images were compared for surface changes.

**Results:**

Through qualitative analysis of the images, there was wear on the enamel surface and all groups, but the G5 worn with smaller specimens equivalent to 22.2%. G2 specimens showed the most wear surface with about 90%.

**Conclusion:**

All evaluated toothpastes showed potential for abrasive wear, but the G2 group showed a higher percentage of worn specimens.

**Clinical relevance:**

The use of whitening toothpaste can cause significant enamel wear, which varies according to the formulation. Therefore, the dentist should guide product selection to balance aesthetics and preservation of tooth structure.

## Introduction

Dental enamel is the most mineralized structure in the human body and defines the shape of the tooth. It is primarily composed of hydroxyapatite, along with proteins and water, making it a hard yet brittle tissue [1]. Enamel is also responsible for the translucency of the tooth, a critical feature for the aesthetic appearance of the smile, which is highly sought after. Despite its properties that confer hardness and translucency, numerous factors can compromise enamel structure, leading to its wear [1].

Enamel wear can lead to both intrinsic and extrinsic alterations, such as changes in tooth color, surface cracks, sensitivity, and pain. These effects may be triggered by the consumption of acidic or pigmented foods and using toothpastes containing abrasive elements or substances that can compromise the mineralized enamel structure [2, 3].

Toothpaste formulations contain several components, including humectants, flavoring agents, preservatives, detergents, abrasives, among others [4]. However, the main concern lies in the continuous use of abrasive agents, which, although effective in cleaning and removing stains from enamel, may also cause structural damage to it [3]. Among the most common abrasive components found in toothpastes are precipitated calcium carbonate, dicalcium phosphate, tricalcium phosphate, aluminum oxide, calcium pyrophosphate, and silica [5]. These abrasives are inorganic, water-insoluble salts that help remove debris from the tooth surface and prevent the formation of stains or pigmented biofilms [6].

The dental market offers a wide variety of whitening toothpastes, which has raised concerns due to the growing societal emphasis on achieving a whiter smile. Consequently, numerous studies have examined the types of components found in so-called whitening toothpastes and the effects of their abrasive agents on dental surfaces [7].

The degree of enamel wear resulting from toothpaste abrasiveness depends on several factors, including the concentration and type of abrasive agent used [3]. Therefore, it is essential to assess both the composition of dentifrices and their potential effects on dental tissues, particularly enamel surface wear [5, 6]. Enamel surface loss is not solely associated with the abrasive type, but is also influenced by various other factors, such as the particle size, concentration, brushing force, and brushing technique [7]. Regarding toothpastes, there is an index that measures their abrasiveness, the Relative Dentin Abrasivity (RDA), which quantifies the abrasive capacity of toothpastes to remove stains and dental plaque from tooth enamel without causing excessive wear [8]. Toothpastes with an RDA below 100 are considered safe for daily use, whereas those with values between 100 and 250 are more abrasive and should be used with caution [9]. Therefore, it is necessary to evaluate how the wide variety of commercially available dentifrices influence the surface characteristics of new materials on the market.

Thus, Optical Coherence Tomography (OCT) serves as a valuable tool for analyzing the microstructure of dental enamel. It is a non-invasive technique that enables the visualization of internal structures in a non-destructive manner [10]. OCT has proven to be highly effective in capturing detailed images of dental tissues for research purposes. OCT uses low-coherence interferometry to produce a two-dimensional image of optical scattering from internal microstructural tissues, analogous to pulse-echo ultrasound imaging. In Dentistry, it has been used to investigate caries, artificial demineralization, and the remineralization of enamel and dentin [11]. This method produces cross-sectional images of biological tissues at micrometer resolution by measuring the time delay of backscattered light according to the depth of the tissue [12].

That way, the lack of non-destructive, imaging-based evaluations of enamel wear caused by whitening toothpastes. While previous studies have assessed toothpaste abrasivity using destructive or surface-limited methods, limited evidence is available using optical coherence tomography (OCT) to qualitatively detect enamel surface changes. Therefore, the aim of this study is to evaluate enamel wear after the use of different toothpastes containing abrasive components, using Optical Coherence Tomography (OCT). The null hypothesis (H_0_): no statistically significant difference in enamel wear between the different whitening toothpaste evaluated and the control group (distilled water).

## Methodology

This work is an experimental laboratory study approved by the Ethics Committee on the Use of Animals of the Center for Biological Sciences at the Federal University of Pernambuco (CEUA-CCB/UFPE), under protocol number 23076.012455/2015-8 (Appendix A). The research was conducted at the Clinical Research Center in Biomaterials at UFPE, in the Photonics and Biophotonics Laboratory of the Department of Physics at UFPE, and in the Biomechanics Laboratory at the School of Dentistry of Pernambuco (FOP/UPE).

A pilot study was conducted to calibrate the procedures and verify the methodological feasibility.

### Specimen selection and preparation

For the experimental phase of this study, bovine incisors were obtained from young bovines, sourced from the Municipal Slaughterhouse of Tupanatinga – PE. The teeth were stored in a 2% chlorhexidine solution for 12 h for disinfection and subsequently kept in distilled water under refrigeration for a maximum period of six months, as recommended by ISO TR-11,405 [13]. Prophylaxis was performed manually using #12 scalpel blades to remove any remaining periodontal ligament, followed by polishing with rubber cups and a slurry of pumice and water.

The specimens were then visually examined under a stereoscopic magnifying lens to assess the integrity of the enamel surface. Any specimens presenting defects or fractures in the enamel were excluded from the study. After this evaluation, the selected teeth were stored in distilled water and refrigerated until further use.

The following materials were selected for this study: four commercial toothpastes—Oral-B 3D White, Colgate Total 12 Gel Whitening, Colgate Luminous White, and Closeup White Now—along with distilled water, a soft-bristled toothbrush (Oral-B Indicator Plus 35), and artificial saliva composition (NaCl − 8.0 g/L, KCl − 0.2 g/L, CaCl₂·2 H₂O -0.2 g/L, MgCl₂·6 H₂O − 0.1 g/L, NaH₂PO₄·2 H₂O − 0.05 g/L, NaHCO₃ − 1.0 g/L, urea − 1.0 g/L and mucin − 2.0 g/L, adjusted to pH 6.8–7.0).

**Fig. 1:**
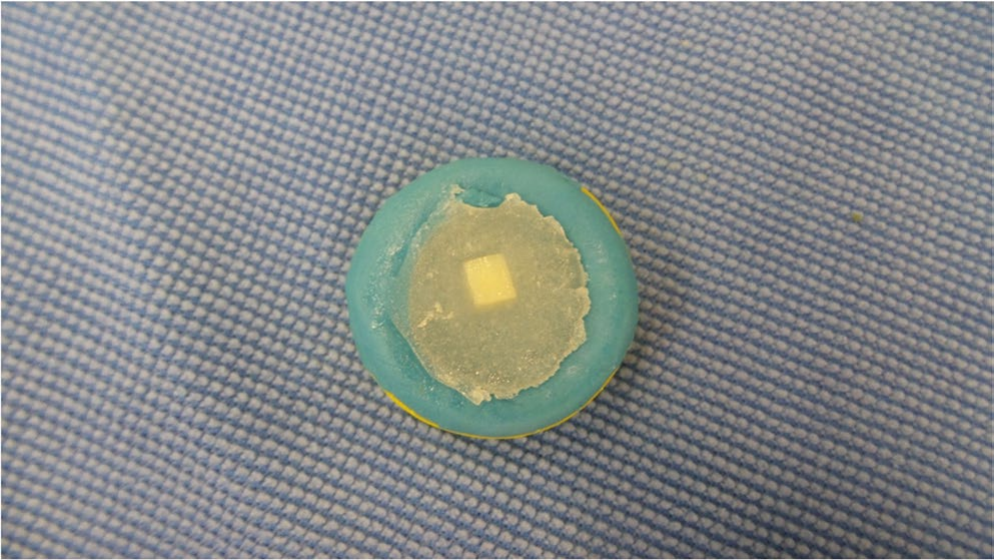
Specimen fabricated

The teeth were sectioned on the buccal surface in both mesiodistal and incisocervical directions using a double-sided diamond disc (KG Sorensen, São Paulo, Brazil) under constant water irrigation, producing enamel fragments measuring 4 mm × 4 mm, a middle third of the central incisors was used for analysis. These fragments were then embedded in chemically activated acrylic resin (JET, Clássico Artigos Odontológicos, Brazil). A mold was fabricated using condensation silicone (COLTENE) with a 5 mm × 5 mm opening. A double-sided adhesive tape was placed on a glass slab, and the enamel fragment was positioned on the tape and inserted into the silicone mold, which was then filled with acrylic resin. This method ensured a flat surface for the test specimens (Fig. 1).

After the resin had polymerized, the buccal surfaces of the specimens were manually polished using aluminum oxide abrasive papers with grit sizes of 600, 1000, and 1200, under continuous water irrigation, followed by polishing with diamond pastes of ¼, 1, 3, and 6 μm particle sizes (Arotec), to obtain a flat and standardized enamel surface.

**Fig. 2:**
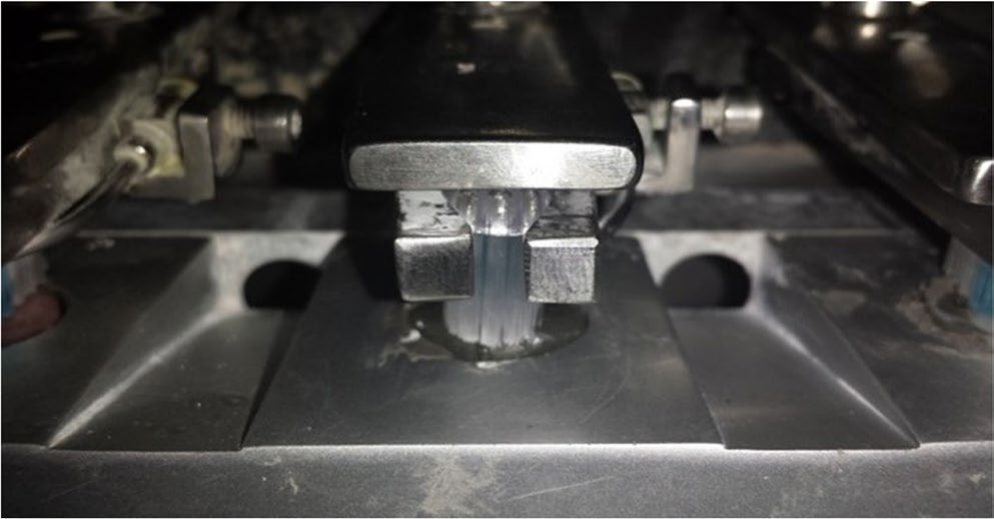
Soft-bristled toothbrushes mounted on the MSET brushing machine

### Experimental group division

The specimens were divided into five groups (*n* = 10) and randomized, according to the toothpaste used, except for Group 5 (G5) (control), which was brushed with distilled water, as described in Table 1. The sample size calculation was based on previous laboratory studies with similar methodology, which evaluated the surface wear of enamel after brushing and the action of bleaching agents. Considering a significance level of 5% (α = 0.05), statistical power of 80% (1 - β = 0.80), and an estimated effect size of Cohen’s d ≈ 1.25, a value considered large for continuous variables in vitro studies, it was estimated that approximately 10 specimens per group would be needed to detect statistically and clinically relevant differences. Thus, *n* = 10 per group was adopted, a number compatible with laboratory studies of similar design and sufficient to ensure adequate analytical power.

**Table 1:** Group division according to the toothpastes used and their respective compositions

GRUPOS	ABREVIAÇÃO	CREME DENTAL	COMPOSIÇÃO
Grupo 1	G1	Oral-B 3D White	Fluoreto de Sódio%(1450 PPM de flúor), Sílica Hidratada, Sorbitol, Larilsulfato de Sódio, Aroma/Flavor, hidróxido de Sódio, água, Sodium Saccarin, Disodium Phhosphate
Grupo 2	G2	Colgate Total 12 Gel Whitening	Fluoreto de Sódio0,32%(1450 PPM de flúor, Triclosano 0,3%, água, sorbitol, Sílica hidratada, copolímero PVM/MA, Larilsulfato de Sódio, Hidróxido de Sódio.
Grupo 3	G3	Colgate Luminous White	Fluoreto de Sódio 0,23% (1100 ppm de flúor), água, sílica hidratada, sorbitol, glicerina, trifosfato de pentasodio, PEG-12, Pirofofato de tetrapotasio, larilsulfato de sódio, hidróxidp de sódio, goma de celulose, sacarina sódica, dióxido de titânio
Grupo 4	G4	Closeup White Now	Fluoreto de Sódio 1450 ppm de flúor, sorbitol, água, Hidrated Silica, PEG-32, Larilsulfato de sódio, celulose GUM, Trisodium Phhosphate, Sodium Saccarin, PVM/MA, Limonene, sacarina sódica
Grupo 5	G5	Água destilada	Água destilada

### Analysis by optical coherence tomography (OCT)

The specimens were analyzed using Optical Coherence Tomography (OCT), available at the Photonics and Biophotonics Laboratory of the Department of Physics at UFPE, both before and after the brushing procedure. This allowed for the comparison of potential surface alterations on the specimens resulting from the mechanical brushing test.

For quantitative analysis, a pair of images (before and after brushing) was selected for each specimen and superimposed for leveling using the Adobe^®^ Photoshop image editing program. Measurements were then performed using ImageJ (bundled version for Mac) to check for surface wear. Eleven measurements were taken for each selected image (Figure). Calibration was performed in pixels (Px), 1062Px on the horizontal axis (x-axis) and 350Px on the vertical axis (y-axis). The data obtained were grouped into a database entered a Microsoft^®^ Excel spreadsheet and then statistically analyzed.

**Fig. 3:**
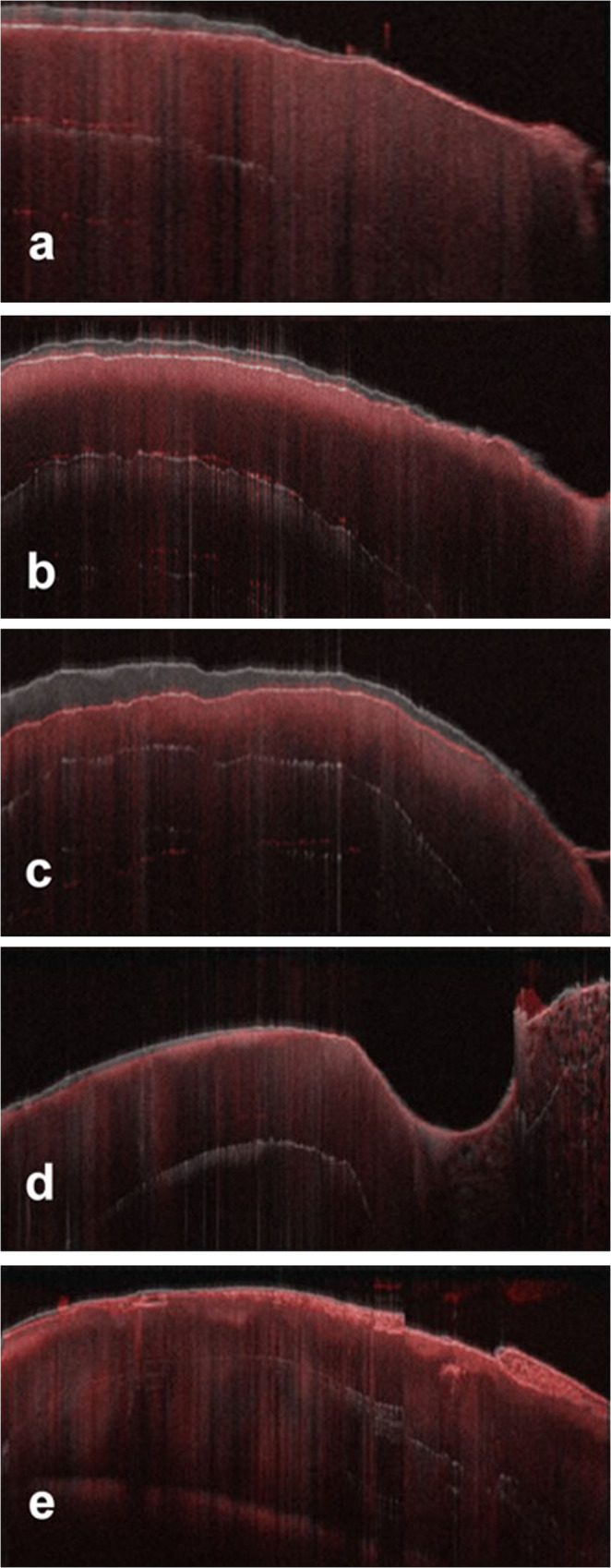
Superimposition of cross-sectional OCT images from different specimens. In image (**e**), no difference in surface level is observed between the images taken before and after brushing. In images (**a**), (**b**), (**c**), and (**d**), a contour below the initial surface level is visible, confirming the occurrence of enamel surface wear following the brushing simulation

OCT employs low-coherence light sources combined with a Michelson interferometer and a system of detectors. The axial resolution is determined by the bandwidth of the light source, while the lateral resolution depends on the optical characteristics of the beam focusing system. The OCT system used in this study was a commercial model (Callisto Spectral Domain OCT System, Thorlabs, USA), equipped with a superluminescent diode light source with a central wavelength of 930 nm, a bandwidth of 10 nm, and a maximum output power of 5 mW. The system’s axial resolution is 7/5.3 μm (in air/water), the lateral resolution is 8 μm, and the maximum penetration depth is up to 1.6 mm. The scanning frequency is 1.2 kHz, capturing two images per second with a sensitivity of 105 dB [14].

Two-dimensional images were acquired, each composed of a numerical matrix of 2000 columns and 512 rows. Images were obtained at 250 μm intervals to allow for complete mapping of the specimens. The results were evaluated by a trained operator experienced in OCT image and graph analysis, who was blinded to the group allocation.

**Fig. 4:**
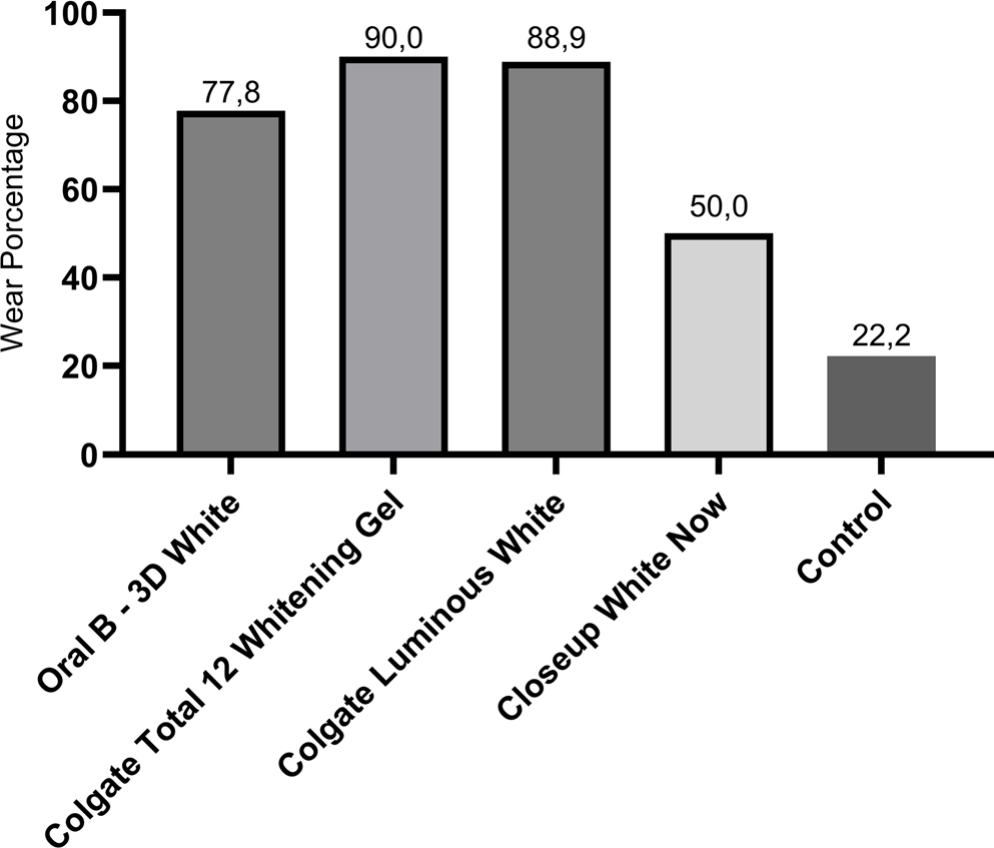
Percentage evaluation of specimens with enamel surface wear by experimental group

### Toothbrushing simulation and specimen storage

Before each brushing cycle, all specimens were immersed in artificial saliva at pH 5.5 for five minutes to simulate the “acid challenge” that typically occurs after food consumption [6]. The mechanical brushing test was conducted using the MSET machine (Elquip, São Carlos, Brazil), located in the Biomechanics Laboratory at FOP/UPE.

Soft-bristled toothbrushes with rounded tips (Oral-B Indicator Plus 35) were mounted on the brushing machine and positioned parallel to the specimen surfaces (Fig. 2). A slurry prepared from each toothpaste (except for Group 5, which was brushed with distilled water only) was used during brushing. The slurries were prepared by mixing each toothpaste with distilled water in a 1:3 ratio, immediately before use [14]. Simulated brushing was performed using linear movements under a static axial load of 200 g at a frequency of 4.5 cycles per second, where each cycle corresponded to a full back-and-forth motion of the toothbrush [1, 5, 15–18].

Each group underwent 5,000 brushing cycles, simulating a total of six months of toothbrushing, with each brushing session lasting approximately 40 min. The brushes were replaced every 1,000 cycles [19, 20].

### Statistical analysis

Three pairs of images (before and after brushing) were selected at equidistant points for each specimen and compared with respect to surface leveling to determine whether enamel wear had occurred.

The data were analyzed descriptively using absolute and relative frequencies, and inferentially using Fisher’s Exact Test. A significant level of 5% was adopted for all statistical tests. The data were analyzed using the Statistical Package for the Social Sciences (SPSS, IBM Corp., Armonk, NY, USA), version 21.

## Results

Figure 3 shows the overlay of OCT images taken before and after the brushing procedure, demonstrating the absence of surface wear (Fig. 3e) and the presence of wear (Fig. 3a, b and c, and 3d). The OCT images captured prior to brushing are displayed in grayscale, while those taken after brushing are shown in red. By overlapping these images, it is possible to identify volumetric changes on the enamel surface, indicating whether wear has occurred.

Tables 2 and 3 present the results related to surface wear of the specimens, according to each experimental group. As shown in Table 2; Fig. 4, the percentage of specimens exhibiting wear was lowest in the Control group (22.2%), followed by the Closeup White Now group (50.0%). The highest percentage was recorded in the Colgate Total 12 Gel Whitening group (90.0%), with values ranging from 7 to 8 in the remaining groups. A significant level of 5.0% was adopted, confirming a statistically significant difference between the groups (*p* < 0.008).

**Table 2:** Avaliação de desgaste da superfície dental segundo o grupo

Grupo	Desgaste de superfície						
	Sim		Não		TOTAL		Valor de *p*
	N	%	N	%	N	%	
Grupo total	**31**	**66**,**0**	**16**	**34**,**0**	**47**	**100**,**0**	
• Oral B – 3D White D	7	77,8	2	22.2	9	100,0	p ^(1)^ = 0,008*
• Colgate Total 12 Gel Whitening	9	90,0	1	10,0	10	100,0	
• Colgate Luminous White	8	88,9	1	11,1	10	100,0	
• Closeup White Now	5	50,0	5	50,0	10	100,0	
• Controle	2	22,2	7	77,8	9	100,0	

(*): Diferença significativa ao nível de 5,0%

(1): Através do teste Exato de Fisher

**Table 3:** Avaliação de amostras com desgaste de superfície entre três imagens avaliadas segundo o grupo

Grupo	Número de amostras com desgaste								
	0		1		2		3		Valor de *p*
	n	% ^(1)^	n	%	N	%	n	%	
Grupo total	**16**	**34**,**0**	**10**	**21**,**3**	**14**	**29**,**8**	**7**	**14**,**9**	
• Oral B – 3D White	2	22,2	4	44,4	2	22,2	1	11,1	p ^(2)^ = 0,006*
• Colgate Total ^12^	1	10,0	-	-	5	50,0	4	40,0	
• Colgate Luminous White	1	11,1	2	22,2	4	44,4	2	22,2	
• Closeup White Now	5	50,0	2	20,0	3	30,0	-	-	
• Controle	7	77,8	2	22,2	-	-	-	-	

(*): Diferença significativa ao nível de 5,0%

(1): Os percentuais foram obtidos com 9 amostras nos grupos Dentrificio Oral B – 3D White, Colgate Total ^12^ e Controle e 10 amostras nos outros dois grupos

(1): Através do teste Exato de Fisher

Table 3 presents the number of specimens with surface wear based on the evaluation of three images per sample within each group. From this table, it is notable that the highest frequency of specimens without wear was observed in the G5 (77.8%), followed by the G4 (50.0%). In the remaining three groups (G1, G2, G3), the number of specimens without wear ranged from one to two. The highest frequency of specimens with two or three instances of surface wear was recorded in the G2, with 9 specimens—this group also showed the highest number of specimens exhibiting wear in all three evaluated images. The second-highest frequency of two or three instances of wear was observed in the G3, with 6 specimens. A statistically significant difference (*p* < 0.006) was found among the groups in terms of the number of specimens with surface wear.

## Discussion

The null hypothesis, which stated that there would be no significant difference in enamel wear among the different whitening toothpastes and the control group, was rejected. The results presented in Tables 2 and 3 demonstrate clear variations in the percentage of worn specimens across the experimental groups. Notably, the Control group exhibited the lowest wear (22.2%), while the Colgate Total 12 Gel Whitening group showed the highest percentage of enamel wear (90.0%). The statistical analysis confirmed a significant difference between groups (*p* < 0.05), indicating that the whitening toothpastes evaluated had varying effects on enamel wear, with some formulations being considerably more abrasive than others.

The use of whitening toothpaste has become an alternative for many individuals seeking to whiten their teeth without professional assistance and at a lower cost. Consequently, concerns have arisen regarding the indiscriminate use of whitening dentifrices and their potential effects on tooth surfaces. Therefore, the aim of this study was to evaluate how these abrasive-containing toothpastes can cause enamel wear through simulated toothbrushing, representing a six-month period of brushing, based on the methodology employed in previous studies [19, 20].

Various instruments are employed to observe the structure of dental enamel, including scanning electron microscopy (SEM), surface profilometry, histological processing with optical microscopy, optical coherence tomography (OCT), among others [4, 18, 21]. In the present study, Optical Coherence Tomography (OCT) was used to analyze dental enamel. This diagnostic method can produce real-time images and providing both qualitative and quantitative data [14]. Numerous studies have used OCT to assess dental structures and have demonstrated its effectiveness in detecting surface alterations in enamel, as observed in this research [12, 21–26].

Among the various changes that can occur in teeth, discoloration over time may result from intrinsic factors during tooth development or from extrinsic factors related to dietary habits. These changes may be classified as extrinsic or intrinsic. Extrinsic discoloration refers to stains on the enamel caused by chromogenic substances such as coffee, chocolate, tobacco, and pigmented beverages, whereas intrinsic discoloration originates from the formation of dental structures themselves or can be acquired through trauma or improper clinical procedures [2].

Given these color alterations that aesthetically compromise the smile, tooth whitening has gained popularity to reduce such discoloration and improve patient comfort. Several techniques have been studied and developed with the aim of removing stains and promoting the whiter smiles that are highly valued today [6]. The abrasiveness of these materials may depend on several factors, including chemical composition, crystal structure, hardness, particle shape, particle size distribution, solubility, concentration, and compatibility with other toothpaste ingredients. It is important to note that these substances are responsible for cleaning tooth surfaces and may contribute to the opacity of the enamel [3].

With the incorporation of abrasive components into dentifrices, numerous studies have been conducted—and continue to be undertaken—to evaluate the wear they may cause on enamel and dentin [1, 4, 5, 18, 25].

Abrasives are crucial for removing dental stains, but they may also lead to surface wear, requiring caution when using dentifrices with high concentrations of such ingredients. According to Martins [27], the main abrasives used include calcium carbonate, hydrated silica, and silicon dioxide, among others. Calcium carbonate, considered one of the most potent abrasives, has been modified to become compatible with fluoride [5]. Silica has been incorporated as a toothpaste abrasive due to several advantages over calcium carbonate, such as being chemically and physiologically inert, odorless and tasteless, having extremely small particles with high absorption capacity, providing excellent product appearance and low density, and offering mild astringent action due to its pH and low abrasivity. Nevertheless, in this study, all tested toothpastes contained hydrated silica.

It is critical to emphasize that brushing abrasivity depends not only on the type of abrasive used in the toothpaste but also on the amount present, as well as the frequency, duration, and force applied during brushing. To minimize these variables, this study employed a brushing machine with standardized load and number of cycles, ensuring that abrasivity was the only factor uniformly analyzed. As a result, the toothpaste that caused the greatest enamel wear was *Colgate Total 12 Gel Whitening* (G2), which contains hydrated silica as its abrasive. However, the specific characteristics of the abrasive were not investigated. As for the other toothpastes, no statistically significant differences were observed among them regarding enamel surface wear. However, when compared to the control group, which was brushed with distilled water, statistically significant differences were identified (Tables 2 and 3). Thus, it is evident that all toothpastes caused enamel surface wear, although Group G2 showed the highest wear percentage.

Most commercial dentifrices do not provide specifications regarding the concentration of abrasives or other ingredients. Often, they also fail to offer guidance on proper usage and contraindications, potentially posing risks to oral health. Greater attention should be given to patients with dental sensitivity, as the frequent use of toothpastes with high abrasive concentrations may worsen their condition [6].

As in the present study, many investigations have employed simulated brushing protocols using various whitening toothpastes, confirming that enamel surface wear is directly related to the presence of different abrasives in the dentifrice, while also emphasizing the need for standardization in applied force and brushing cycles [4, 15, 19–21, 28–30]. The study by Lima [19] evaluated the whitening effectiveness of dentifrices for removing extrinsic stains using toothpastes such as *Colgate*, *Cristal Whitening Extra*, and *Rapid White*, by subjecting specimens to simulated brushing with 5000 cycles. Only *Rapid White* was effective in removing extrinsic stains, which suggests that its higher abrasive content contributed both to enhanced stain removal and greater enamel surface wear.

In this study, all dentifrices that caused enamel wear contained hydrated silica in their composition. However, it is important to note that abrasion is also influenced by the size, distribution, and regularity of the abrasive particles. Larger and more irregular particles tend to cause more wear; therefore, knowing the specifications of the silica abrasive is fundamental [28]. The study by Macedo, Oliveira, and Mercatônio [21] analyzed dentifrices containing both silica and hydrated silica. They observed that toothpaste with silica resulted in greater abrasion compared to hydrated silica. The authors suggested that the lower wear caused by the hydrated silica may be due to its modified form. Differing from the present study, all tested toothpastes here contained hydrated silica as their abrasive.

Despite the limitations inherent to an in vitro experiment, this study demonstrated a statistically significant difference in enamel surface wear caused by toothpaste containing abrasives. However, further research, particularly clinical studies, is needed. It is worth emphasizing that the greater enamel wear observed in Group 2 (90%) is due to the abrasive’s concentration, particle size, or shape. Another limitation is the use of permanent bovine mandibular incisors instead of human enamel, differences in microstructure and hardness may affect clinical extrapolation, but according to Campos et al. (2008) [31], bovine teeth proving to be excellent substitutes for human teeth in dental research due to their ease of delivery and being highly recommended by Ethics Committees. Nonetheless, this study’s methodology did not extend to an in-depth investigation of the specific characteristics of the abrasives, which should be addressed in future research.

## Conclusion

This study has identified that:

- All toothpastes with the abrasive hydrated silica caused wear on the enamel surface.
- Group G2 showed the greatest wear.
- The abrasiveness of brushing does not depend solely on the abrasive present in the toothpaste, but is related to the amount present, frequency, duration, and force during brushing.
- Toothpastes containing silica as an abrasive agent combined with Sodium Lauryl Sulfate potentiate the abrasive action.

## Data Availability

No datasets were generated or analysed during the current study.
